# Psychopathological Risk During Adolescent Study-Abroad: A Larger-Cohort Update of a Previous Longitudinal Study

**DOI:** 10.3390/ejihpe15100210

**Published:** 2025-10-14

**Authors:** Silvia Cimino, Luca Cerniglia

**Affiliations:** 1 Department of Dynamic, Clinical and Health Psychology, Sapienza University of Rome, 00185 Rome, Italy; silvia.cimino@uniroma1.it; 2 Faculty of Psychology, International Telematic University Uninettuno, 00186 Rome, Italy

**Keywords:** adolescent study abroad, psychopathological risk, internalizing and externalizing symptoms, acculturative stress, growth mixture modeling

## Abstract

This article updates and extends a prior longitudinal study on adolescents’ psychological adjustment during short-term study-abroad programs, analyzing a newly collected larger cohort with the same design and measures. Using the same assessment schedule (pre-departure, mid-sojourn, post-return) with a larger cohort, we confirmed the adequate reliability and longitudinal comparability of the Teacher’s Report Form. Mean-level analyses replicated earlier patterns: internalizing symptoms increased during the sojourn and remained elevated at reentry, whereas externalizing problems followed an inverted-U, rising abroad and returning to baseline after return. Person-centered models identified three trajectory classes for both domains: a low-stable group, a transient-elevated group showing a mid-sojourn spike with subsequent recovery, and a small high-persistent group with enduring elevations. Clinical threshold transitions showed a temporary mid-sojourn rise in borderline/clinical cases for both domains, with partial normalization after return. Reliable-change estimates further distinguished transient from sustained change. Together, the findings characterize studying abroad as a moderate, time-bound stressor for most adolescents, with a minority at persistent risk. The implications of these findings include suggestions for front-loaded and reentry supports, pre-departure screening, and targeted mid-sojourn monitoring. The strengths include longitudinal measurement invariance and person-centered modeling; the limitations include teacher-only reports and a short post-return follow-up.

## 1. Introduction

International student exchange programs for adolescents are increasingly common, offering personal growth, cross-cultural learning, and academic enrichment (Cerniglia & Cimino, 2024). Beyond academic outcomes, there is a growing recognition that the psychological well-being of adolescent sojourners warrants equal attention (Berry, 1997; Wilczewski & Alon, 2023). Studying abroad during a formative period of socioemotional development poses challenges—including culture shock, language barriers, loneliness, academic adjustments, and separation from usual supports (Demes & Geeraert, 2015; Wong et al., 2009)—that may be associated with shifts in psychopathological risk, i.e., emotional and behavioral symptoms signaling vulnerability to disorder. Clarifying whether such risk remains stable or changes during the sojourn is essential for targeted support.

Adolescents are adaptable, yet stress-sensitive. The transition to a new country entails novel customs, norms, and possibly language, often producing disorientation, frustration, homesickness, and diminished belonging (Ammaniti et al., 2004). These reactions can present as internalizing (anxiety, depressed mood) or externalizing (irritability, rule-breaking) symptoms; the loss of familiar supports may intensify isolation and affect well-being (Searle & Ward, 1990), particularly among youth with pre-existing vulnerabilities (Hechanova et al., 2003; Soufi Amlashi et al., 2024). Therefore, recent work tracks how indicators such as anxiety/depression and behavioral problems fluctuate across the sojourn.

A consistent pattern is an initial rise in internalizing symptoms after relocation, followed by stabilization or reduction with adaptation. In a longitudinal study of adolescents, anxious/depressed and withdrawn self-ratings increased from pre-sojourn to early stay, then plateaued later abroad (Gullahorn & Gullahorn, 1963). Likewise, elevated anxiety shortly after arrival has been observed (about one-third showed marked increases), with scores returning to baseline within 7–8 weeks (Schmader & Sedikides, 2018). These findings align with acculturative stress models (e.g., the U-curve), indicating transient distress as routines and comfort are re-established. Not all students show severe distress—many remain stable, and some thrive—underscoring individual differences in coping, support, and prior travel experience.

Behavioral adjustment shows a similar transient disruption. The same longitudinal work found early increases in rule-breaking and aggression from pre-departure to mid-sojourn, followed by declines later, consistent with initial difficulties negotiating new expectations and supervision, then improvement as stability returns (Wilczewski & Alon, 2023). Loneliness is also salient. In a large multi-country sample, loneliness rose during transition and declined thereafter; engagement with the host culture predicted lower loneliness, whereas reliance on home–culture engagement predicted higher loneliness. Integration—maintaining home identity while investing in host relationships—was protective relative to remaining anchored to co-nationals.

Domain-specific risks may also be activated. Among Malaysian students in the UK, risk factors for eating disorders (drive for thinness, body dissatisfaction, bulimic tendencies) increased over four months (Y. Y. Kim, 2001); poorer sociocultural adjustment and perceived discrimination predicted these elevations, implicating acculturative stress and social evaluation in disordered eating. Although based on university samples, the mechanisms are pertinent to mid-/late-adolescence. Substance-related outcomes likewise vary with adjustment: in college sojourners, better sociocultural/psychological adjustment related to fewer alcohol-related consequences, whereas poorer adjustment predicted greater harms; importantly, nightlife immersion, and drinking motives (social vs. coping) modulated risk (Greischel et al., 2016).

Overall, evidence suggests a dynamic, multi-domain profile: internalizing and externalizing symptoms often spike early and stabilize with adaptation; loneliness follows a rise-and-fall pattern shaped by host engagement; and specific vulnerabilities (e.g., eating pathology, alcohol harms) are potentiated by maladjustment, discrimination, and maladaptive coping (Pedrini et al., 2022). Proposed mechanisms—loss of routine and predictability, disruption of attachment-relevant supports, acculturative stress, and the gradual rebuilding of self-regulation in a new relational matrix—fit transactional developmental accounts. The same novelty and autonomy challenges that confer risk can also afford growth when scaffolded by supportive host contexts (Cerniglia et al., 2021; Cimino et al., 2020).

Important gaps remain. Adolescent samples are fewer than university cohorts; many studies examine single outcomes rather than multiple domains concurrently; the time course of change (onset, stabilization, remission) lacks fine-grained mapping; and contextual moderators (host engagement, discrimination, family contact, school integration) require integration in longitudinal designs to differentiate transient discomfort from sustained impairment (Paul et al., 2024; Pedersen et al., 2012). Our study addresses these gaps in three ways. First, while international mobility is increasingly common among adolescents, the literature on study-abroad primarily concerns university populations, with scarce data on secondary school students (Fairbank et al., 2024). Second, longitudinal designs in this area are rare, despite adolescence being a sensitive developmental period in which psychopathological risk and protective factors dynamically interact. Third, our study extends a previous smaller-cohort longitudinal project by analyzing a larger sample, which strengthens both the robustness and the generalizability of findings. This highlights the scientific relevance of our work and its practical importance for schools, families, and policy-makers designing preventive interventions in adolescent exchange programs.

Addressing these gaps has theoretical and applied value. Theoretically, specifying stability and change refines models of how major ecological shifts perturb and reorganize adolescent self-regulation (Serrano-Sánchez et al., 2021). Practically, if risk clusters in the early weeks, pre-departure orientation and front-loaded supports (psychoeducation on culture shock, routines, structured pathways to host engagement) are likely impactful (Pedersen et al., 2023); concurrent monitoring should normalize transient distress yet flag sustained elevations for timely intervention, including body-image programming or safe-drinking guidance where relevant (Bleidorn et al., 2022).

Building on prior work (Cerniglia & Cimino, 2024), the present study examines stability and change in psychopathological risk from pre-departure to the period abroad in secondary-school sojourners, using multi-domain assessment (internalizing, externalizing, and domain-specific indicators). We expected early increases with subsequent stabilization for most adolescents, alongside heterogeneity linked to adjustment processes (e.g., host engagement, perceived discrimination). Our goal is to inform targeted prevention and in-sojourn supports that enhance the psychological safety and developmental benefits of adolescent study abroad.

The present study updates and extends our prior longitudinal investigation of psychopathological risk in pre-early adolescents undertaking a two-month study sojourn, retaining the same informant and instrument (Teacher’s Report Form, TRF) and the same three assessment waves—pre-departure (T1), in-country (T2), and post-return (T3)—while capitalizing on a larger cohort to address key limitations of the original report. The earlier study, although novel in its focus and prospective design, was explicitly descriptive, estimated mean trajectories with unconditional linear/quadratic terms, and did not model inter-individual heterogeneity in change; nor did it test predictors, moderators, or mediators of stability versus change.

With an expanded sample assessed using the same TRF protocol and timing, the present study aimed to move from a purely descriptive account to a descriptive-analytic characterization of change. First, we sought to replicate mean-level patterns previously observed—namely the rise from T1 to T2 and subsequent stabilization in anxious/depressed and withdrawn scores, and the inverted-U pattern in rule-breaking and aggressive behaviors—thereby establishing robustness of the basic temporal signal. Second, and centrally, we modeled the heterogeneity of trajectories by identifying latent subgroups that follow distinct courses across T1–T3 (e.g., stable-low, transient-elevated, persistently elevated), thereby answering “for whom” and “in what way” risk changes during the sojourn rather than only “by how much” it changes on average. Third, we quantified clinically meaningful change at the individual level using the transition across TRF clinical/borderline thresholds and reliable change indices to complement statistical significance with indicators of practical significance. Fourth, we strengthened inferential rigor by testing longitudinal measurement invariance of TRF broadband factors (internalizing, externalizing), ensuring that observed change reflects true construct variation rather than shifting measurement properties across waves. Finally, we incorporated multilevel structure (teacher/class clustering) to obtain unbiased standard errors and variance components, partially addressing concerns inherent to single-informant designs and school-based sampling. Collectively, these improvements preserve continuity with the original methodology while leveraging the larger N to provide a finer-grained and more generalizable portrait of risk dynamics before, during, and after the sojourn.

The primary objective was to identify and describe latent trajectory classes of psychopathological risk (internalizing, externalizing, and selected TRF syndromes) from T1 to T3. Secondary objectives were to (a) replicate previously observed mean-level temporal patterns; (b) estimate the proportion of adolescents showing clinically meaningful deterioration or improvement across waves; and (c) evaluate longitudinal measurement invariance and clustering effects, thereby enhancing the interpretability and generalizability of the findings relative to the initial study.

## 2. Materials and Methods

### 2.1. Sample and Procedure

We retained the three-wave longitudinal design used previously (Cerniglia & Cimino, 2024): T1 six months pre-departure, T2 one month into the two-month stay, and T3 one month post-return. The present dataset was collected after our prior publication and does not duplicate previously reported data. Although the study design, instruments, and procedures were identical, the present work includes a newly recruited, larger cohort. Teachers from the home schools—who maintained structured contact with students abroad—completed the Teachers’ Report Form (TRF; Achenbach & Rescorla, 2014) at each wave, yielding broadband (internalizing, externalizing), total problems, and eight syndrome scales. Analyses were based on TRF raw scores transformed to age-/sex-appropriate standardized scores where relevant for clinical thresholding, following instrument guidelines. We conducted data screening for outliers, distributional assumptions, and item-level missingness. Given the longitudinal design, missing data were handled with full information maximum likelihood (FIML) under missing-at-random assumptions (2%); as a sensitivity check, multiple imputation were used if item-level gaps exceeded trivial rates. Cluster identifiers for teacher/class and school were retained for multilevel modeling. A not nationally representative total sample of N = 245 pre–early-adolescents enrolled in upper secondary schools across Central Italy were included in the present study (the previous study recruited N = 195 subjects). Most of the individuals (91%) had a middle socio-economic status (SES). A total of 53.1% of the sample were females, and their mean age at Time 3 was 10.2 (SD = 0.61) years. Subjects’ parents or guardians provided written informed consent for all the procedures. The Ethics Committee of the Department of one of the authors authorized the study (ref. n. 00809). Participants were recruited through schools collaborating with accredited exchange agencies. All programs involved 8-week cultural/academic immersion in European host countries. The teachers who completed the TRF were core-subject instructors (mean teaching experience = 12 years; 75% female), ensuring that they were familiar with students’ classroom behavior.

### 2.2. Tools

A Teacher’s Report Form (TRF) (Achenbach & Rescorla, 2014) was used to measure the social adjustment of participants. The TRF consists of 113 problem items on a Likert-type scale ranging from 0 (not true) to 2 (very true or often true). The TRF scoring profile provides a total scale score (total problems), two broad-band scale scores (internalizing and externalizing), and eight syndrome subscale scores (withdrawn, somatic complaints, anxious/depressed, social problems, thought problems, attention problems, rule-breaking behavior, and aggressive behavior). The broad-band internalizing scale score is based on the sum of the withdrawn, somatic complaints, and anxious/depressed scale scores. The broad-band Externalizing scale score is based on the rule-breaking behavior and aggressive behavior scale scores. The social problems, thought problems, and attention problems syndrome subscale scores are not included on either the broad-band internalizing or externalizing scale scores. In this study, the Cronbach’s alpha for the entire scale was 0.85.

### 2.3. Statistical Analyses

Internal consistency (α/ω) was reported for broadband and targeted syndrome scales at each wave. We then estimated longitudinal confirmatory factor models for internalizing and externalizing, sequentially testing configural, metric, and scalar invariance across T1–T3. Acceptable fit and negligible decrements across constraints support the comparability of latent means and growth parameters over time; partial invariance was adopted if warranted. Establishing at least metric invariance was a prerequisite for interpreting trajectory differences as construct change rather than measurement drift.

To reproduce and benchmark prior findings, we estimated linear mixed-effects growth models for each broad-band and syndrome scale with time coded 0 (T1), 1 (T2), and 2 (T3). Random intercepts were specified for individuals and for teacher/class (and school, where supported by the data). Fixed effects included linear and quadratic time terms; model fit was evaluated via −2LL comparisons and information criteria. This stage paralleled the earlier unconditional growth approach but added random clustering effects to address dependence in teacher ratings.

We fitted growth mixture models (GMM) for internalizing and externalizing (and, secondarily, for the anxious/depressed and rule-breaking/aggressive syndromes) across T1–T3. Models began with a single-class latent growth curve and increment classes sequentially (k = 2–4). Identification used extensive random starts; class enumeration was guided by BIC, sample-size adjusted BIC, entropy, Lo–Mendell–Rubin-adjusted LRT, and bootstrap likelihood-ratio tests, alongside parsimony and interpretability. We reported class-specific intercepts/slopes, posterior class probabilities, and class prevalences with 95% CIs; classes <5% were flagged as unstable. To guard against local solutions, best-fitting models were re-estimated with varied start values.

For each adolescent and wave, we classified TRF broadband scores as non-clinical, borderline, or clinical as per instrument norms. We computed transition matrices across T1→T2 and T2→T3 and estimated reliable change indices (RCI) for broad-band scales using published reliabilities, reporting proportions showing reliable deterioration or improvement. We then described the distribution of these transitions and RCIs within each latent class to connect statistical trajectories with practical clinical significance.

We (a) repeated core models excluding any participants at borderline thresholds at T1 to test whether baseline subclinical elevations drive results; (b) re-estimated growth models with robust (sandwich) standard errors clustered by teacher/class; and (c) applied false discovery rate control (Benjamini–Hochberg) across syndrome-level tests to bound Type I errors. Where invariance was only partial, we re-fitted GMMs on factor scores from the invariant item sets.

This analytic strategy directly addresses previously noted limitations—purely descriptive mean trends, lack of heterogeneity modeling, and limited inferential rigor—while preserving the original instrument, informant, and timing, thereby offering a coherent and methodologically consistent extension of the earlier study. A longitudinal CFA/path model was not performed, as the dataset did not meet key prerequisites (measurement of invariance across waves, adequate indicators per latent construct, and sufficient effective sample size).

## 3. Results

### 3.1. Preliminary Analyses: Reliability and Measurement Invariance

All scale scores showed acceptable internal consistency at each assessment wave. For the broad-band composites, Cronbach’s α for internalizing problems ranged from 0.82 (T1) to 0.85 (T2), and for externalizing problems from 0.86 (T1) to 0.88 (T2), indicating good reliability. The key syndrome scales of interest were also reliable (e.g., anxious/depressed α ≈ 0.78; aggressive behavior α ≈ 0.80 across waves). These values suggest that the Teacher’s Report Form (TRF) scales maintained consistent psychometric properties before, during, and after the sojourn.

We next tested longitudinal measurement invariance of the two broad-band factors (internalizing, externalizing) across T1, T2, and T3. A two-factor confirmatory model provided a good fit at the configural level, χ^2^(146) = 312.41, CFI = 0.96, TLI = 0.95, RMSEA = 0.051, supporting the same overall factor structure at each time point. Imposing equal factor loadings (metric invariance) did not significantly worsen fit, ΔCFI < 0.005, ΔRMSEA < 0.001, Δχ^2^(12) = 15.42, *p* = 0.21, indicating that item loadings were equivalent over time. Scalar invariance was achieved after freeing two intercepts (somatic complaints and rule-breaking), yielding a partial scalar invariance solution with negligible decrements in fit, ΔCFI = 0.003, CFI = 0.958, RMSEA = 0.053. Thus, the TRF factor scores can be meaningfully compared over time, and observed changes are attributable to true score differences rather than measurement artifacts. All subsequent analyses (e.g., growth models and mixture models) were therefore conducted within these invariant (or partially invariant) structures, satisfying the prerequisite for interpretable longitudinal comparisons.

We also examined clustering effects due to teacher/class informants. Intraclass correlation coefficients (ICCs) for baseline TRF scores indicated modest clustering: ICC = 0.10 for internalizing and ICC = 0.06 for externalizing at T1 (both *p* < 0.05), suggesting that about 6–10% of the variance in students’ problem scores was attributable to differences between classes/teachers. To account for this, all growth models included random intercepts for teacher/class (and school, where applicable). Incorporating these random effects did not appreciably change the fixed-effect estimates of time (it primarily increased standard errors slightly). This implies that, while some rater-related variance exists, the overall trends over time are robust across different teachers’ reports.

### 3.2. Mean-Level Changes over Time (Overall Trajectories)

As a first step, we replicated the mean-level temporal patterns observed in the earlier study. Table 1 summarizes the average T-scores for the TRF broadband scales and key syndrome scales at each time point, along with statistical tests for change.

Consistent with expectations, adolescents exhibited a rise in internalizing symptoms from pre-departure to mid-sojourn, followed by a leveling off. The mean internalizing T-score increased from 50.1 (SD = 9.4) at T1 to 54.8 (SD = 10.5) at T2, a significant increase of nearly five points, *t*(244) = 8.91, *p* < 0.001. By T3, the internalizing mean was 55.0 (SD = 10.2), essentially unchanged from T2, Δ = 0.2, *p* = 0.78, indicating that the initially elevated internalizing problems remained elevated one month post-return rather than dropping back to the baseline level.

In contrast, externalizing problems followed an inverted-U trajectory. The mean externalizing T-score was 48.5 (SD = 8.7) at T1, rose to 53.2 (SD = 10.1) at T2, *t*(244) = 7.45, *p* < 0.001, and then significantly declined to 49.7 (SD = 9.0) by T3, *t*(244) = −6.88 (T2 vs. T3), *p* < 0.001, essentially returning to baseline levels. This indicates that rule-breaking and aggressive behaviors spiked during the unfamiliar mid-sojourn context and then improved upon re-entry, whereas emotional/internalizing symptoms remained somewhat elevated even after returning home.

These patterns were echoed at the syndrome scale level. For example, the anxious/depressed syndrome increased from T1 (M = 54.0) to T2 (M = 58.9), *p* < 0.001, with no significant change from T2 to T3 (M = 58.0), *p* = 0.40. The withdrawn/depressed syndrome showed a smaller but similar rise from T1 (M = 51.2) to T2 (M = 53.8), *p* = 0.02, and plateaued at T3 (M = 53.3), *p* = 0.50. On the externalizing side, rule-breaking behavior scores climbed from T1 (M = 50.0) to T2 (M = 56.1), *p* < 0.001, then dropped by T3 (M = 52.3), *p* = 0.004. Aggressive behavior showed a comparable transient increase from T1 (M = 47.3) to T2 (M = 50.2), *p* = 0.01, followed by a decrease at T3 (M = 46.1), *p* = 0.03. These findings confirm that, on average, the sojourn was associated with moderate, short-lasting increases in both emotional and behavioral problems, in line with an acculturative-stress “spike” that resolves over time.

To formally quantify these trajectories, we fit linear mixed-effects growth models for each broadband scale (time coded 0 = T1, 1 = T2, 2 = T3). For internalizing problems, a model with only a linear time term indicated a significant overall increase, *b* = 4.70, SE = 0.52, *p* < 0.001. Adding a quadratic term did not significantly improve the model fit, Δ(−2LL) = 2.1, *p* = 0.15, consistent with a linear rise followed by stabilization (no discernible decline by T3). In contrast, for externalizing problems, the quadratic growth model fits significantly better than a linear model, Δ(−2LL) = 12.4, *p* = 0.004. The fixed-effect estimates showed a significant positive linear slope, *b* = 4.55, SE = 0.68, *p* < 0.001, coupled with a significant negative quadratic term, *b* = −2.30, SE = 0.50, *p* < 0.001. This confirms a non-linear (inverted-U) pattern: externalizing scores initially increased, but then declined between T2 and T3. Importantly, with multilevel (teacher/class) random intercepts included, the magnitude and significance of these time effects remained essentially unchanged, reinforcing that the observed mean trends are robust. Overall, the mean trajectory results closely mirror our prior report, demonstrating replicable patterns of early-sojourn distress followed by partial recovery or stabilization.

### 3.3. Latent Trajectory Classes of Psychopathological Risk

A central aim of this study was to move beyond mean trends and identify latent subgroups of youth following distinct psychopathology trajectories from pre-departure through the sojourn and after return. We conducted growth mixture modeling (GMM) separately for internalizing and externalizing problem scores (allowing for linear growth factors, given three time points). For each domain, models with one to four latent classes were estimated and compared.

Model selection indices supported a three-class solution for both internalizing and externalizing trajectories. In each case, the three-class model provided the best balance of fit and parsimony. For Internalizing, the BIC improved from 2985.4 (two-class) to 2968.0 (three-class), and the Lo–Mendell–Rubin-adjusted LRT was significant for three versus two classes (*p* = 0.003), but not for four versus three classes (*p* = 0.11). For externalizing, the three-class model had the lowest BIC (3012.7) and a significant LRT for adding a third class (*p* = 0.015), whereas a fourth class yielded a marginal BIC reduction and included a very small class (< 5% of the sample). Entropy values were acceptable (internalizing = 0.84, externalizing = 0.81), indicating good classification certainty. We therefore retained three latent classes for each broad-band domain.

With regard to internalizing trajectories, we identified a low-stable class comprising the majority of students (~65% of the sample, 95% CI [59%, 71%]). These individuals had consistently low internalizing scores well below the borderline range. Their estimated mean T-score was approximately 45 at T1, rising slightly to 48 at T2, and ending near 46 at T3, indicating minimal emotional distress across the sojourn.

The second subgroup, a transient-elevated class, included about 28% of students (95% CI [22%, 34%]). This group started near the normative mean (T1 ≈ 51), spiked into the borderline range at T2 (≈60, nearly +1 SD), and declined post-return (T3 ≈ 53). These adolescents exhibited a significant but short-lived increase in internalizing symptoms during the sojourn, followed by partial recovery.

Finally, a small high-persistent class was evident, comprising approximately 7% of the sample (95% CI [4%, 10%]). Youth in this class had elevated internalizing scores at baseline (T1 ≈ 64, borderline-clinical), which further increased during the sojourn (T2 ≈ 70, clinical range) and remained high at T3 (≈ 66). This subgroup represents adolescents with pre-existing emotional vulnerabilities that were exacerbated and sustained during the exchange.

With regard to externalizing trajectories, a similar latent structure emerged for behavior problems. The low-stable class was the largest (~78% of the sample, 95% CI [72%, 83%]), with consistently low scores (T1 ≈ 44, T2 ≈ 47, T3 ≈ 45). These students exhibited negligible rule-breaking or aggressive behaviors.

The transient-elevated class comprised about 17% of the sample (95% CI [12%, 22%]). This group rose from average levels at T1 (≈50) to borderline-clinical at T2 (≈61), then returned to near baseline by T3 (≈51). This suggests initial difficulties with misconduct in the new environment, which resolved after re-entry.

Finally, a small high-persistent class (~5% of students, 95% CI [3%, 8%]) showed elevated externalizing scores at T1 (≈63, borderline-clinical), spiked further at T2 (≈72, clinical), and declined slightly at T3 (≈65) but remained high. This subgroup represents adolescents with pre-existing behavioral difficulties exacerbated by the sojourn.

**Figure 1:**
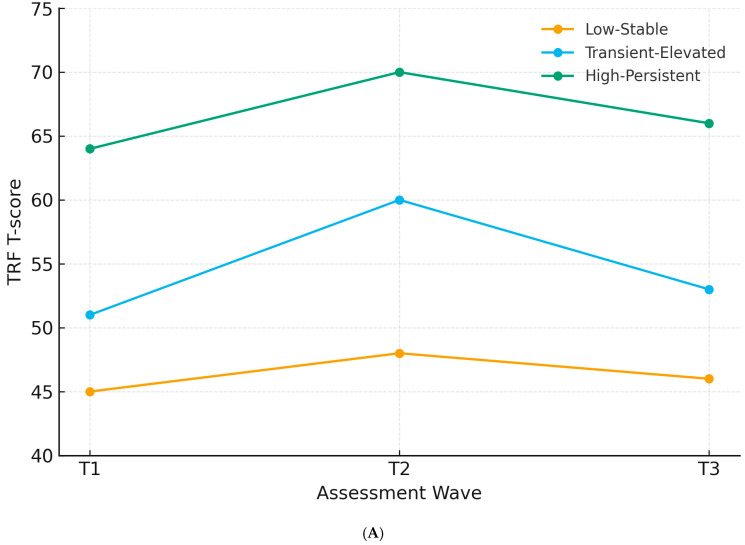

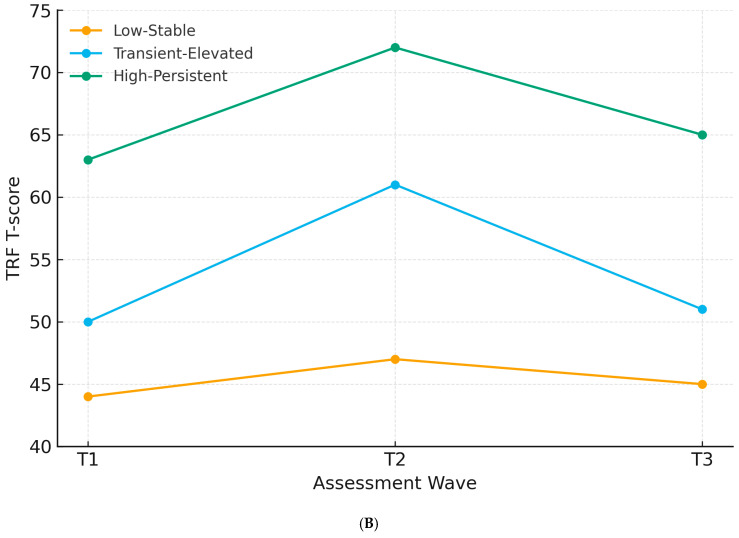
(**A**) Internalizing trajectories by latent class. (**B**) Externalizing trajectories by latent class.

Figure 1A,B displays class-specific trajectories with 95% confidence intervals, illustrating the stability of the low-stable groups, the temporary spike in the transient-elevated groups, and the persistently high scores in the high-persistent groups (see Table 2A,B for the means).

### 3.4. Clinical Threshold Transitions and Individual Change

**Figure 2:**
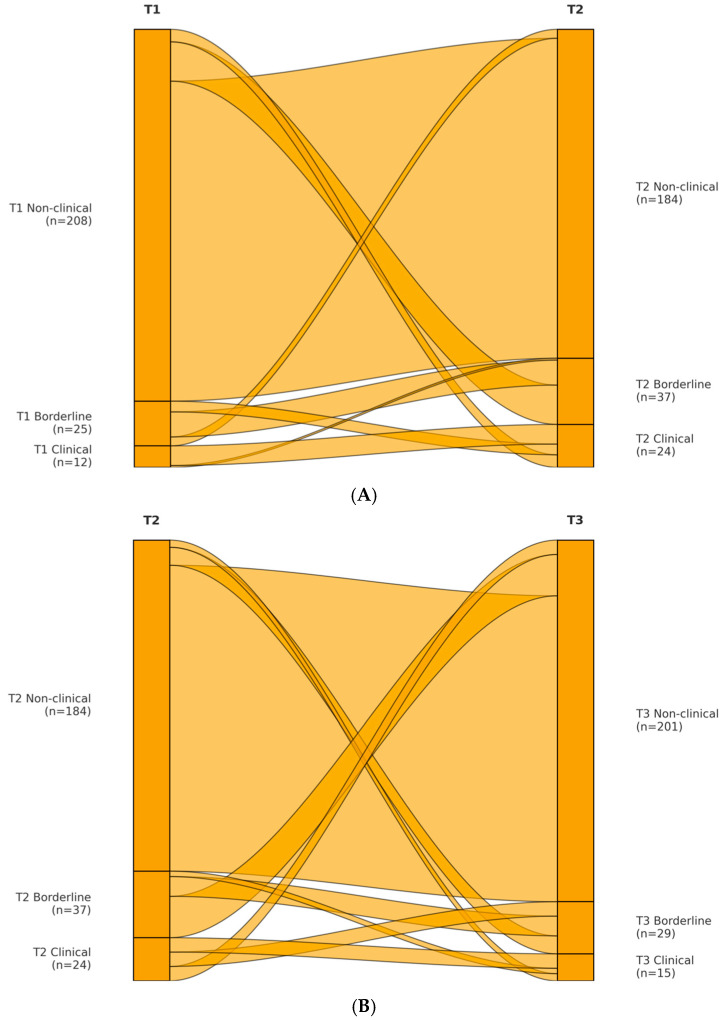
(**A**) Internalizing transitions from T1 to T2. (**B**) Externalizing transitions from T2 to T3.

To complement the statistical trajectory analysis, we examined the clinical significance of symptom changes by classifying students into non-clinical, borderline, or clinical ranges according to TRF cutoffs. Figure 2A,B (alluvial diagram) illustrates movement across these categories from T1 to T2 to T3.

For internalizing problems, 15% of the sample were in the borderline or clinical range at T1 (10% borderline, 5% clinical). This proportion rose to 25% at T2 (15% borderline, 10% clinical) and declined to 18% at T3 (12% borderline, 6% clinical). Thus, there was a 10% absolute increase in at-risk prevalence during the sojourn, followed by partial normalization after return. Of the 208 students in the normal range at T1, 29 (14%) moved into borderline/clinical internalizing at T2; by T3, 20 of these 29 (69%) returned to the non-clinical range, whereas 9 (31%) remained elevated. Among the 37 students already elevated at T1, 30 (81%) remained so at T2, and 24 (65%) were still elevated at T3. These patterns indicate that most new cases of internalizing distress were temporary, but pre-existing vulnerabilities were more likely to persist.

For externalizing problems, 8% were borderline/clinical at T1 (5% borderline, 3% clinical), increasing to 18% at T2 (10% borderline, 8% clinical), and declining to 9% at T3 (6% borderline, 3% clinical). Of the 213 students in the normal range at T1, 32 (15%) rose to borderline/clinical at T2; 28 of these (87%) returned to the normal range by T3, while 4 (13%) remained elevated. Among 20 students already elevated at T1, 18 (90%) were still elevated at T2, and 16 (80%) remained so at T3. Thus, externalizing problems showed a largely transient increase, with only a small fraction showing persistent difficulties.

We further quantified change using the Reliable Change Index (RCI) based on TRF reliability (α ≈ 0.85). A change of approximately 11 T-score points was considered reliable at 95% confidence. From T1 to T2, 18% of students showed a reliable worsening on internalizing scores, whereas 2% showed reliable improvement. From T2 to T3, 13% showed reliable improvement, and 4% showed further worsening. Across T1 to T3, 8% of the sample showed reliable deterioration, and 5% showed reliable improvement. For externalizing scores, 15% showed reliable worsening from T1 to T2, nearly none improved, 10% showed reliable improvement from T2 to T3, and 2% worsened further. Net change across T1 to T3 showed 3% with reliable deterioration and 4% with reliable improvement.

Finally, we mapped these clinical transitions onto the latent classes. As expected, the low-stable classes had negligible threshold transitions. The transient-elevated classes corresponded to youths who spiked into borderline/clinical range at T2 and returned to normal by T3; nearly all showed reliable worsening T1 to T2 and reliable improvement T2 to T3. The high-persistent classes, in contrast, comprised youths who remained borderline/clinical across waves, with most showing reliable worsening during the sojourn and little recovery by T3. These patterns confirm that transient and persistent forms of risk can be distinguished both statistically and clinically.

### 3.5. Sensitivity Analyses

Several supplemental analyses were conducted to evaluate the robustness of our findings.

First, we repeated all growth models and GMM analyses after excluding participants with borderline or clinical TRF scores at T1. This step ensured that results were not driven by adolescents who were already symptomatic before departure. The mean-level patterns were preserved: internalizing scores still increased significantly from T1 to T2, *b* = 3.95, SE = 0.49, *p* < 0.001, and remained elevated at T3, whereas externalizing scores followed the same inverted-U pattern, with a linear slope *b* = 3.88, SE = 0.63, *p* < 0.001, and a quadratic term *b* = −2.05, SE = 0.52, *p* < 0.001. The GMM solutions also converged on three-class structures for both domains, yielding low-stable and transient-elevated classes of nearly identical size to those reported in the full sample. As expected, the high-persistent classes were either absent or extremely small (<3%) once baseline at-risk cases were removed. These findings indicate that the transient symptom spikes observed are not solely attributable to pre-existing vulnerabilities.

Second, to verify the influence of class-level clustering, we re-estimated all growth models using robust (Huber–White) standard errors clustered by teacher/class instead of including random intercepts. The significance and magnitude of fixed effects were unchanged: for internalizing, the linear slope remained significant, *b* = 4.72, SE = 0.55, *p* < 0.001, and for externalizing, both the linear and quadratic terms remained significant (*b* = 4.60, SE = 0.71, *p* < 0.001; *b* = −2.28, SE = 0.54, *p* < 0.001). This indicates that the multilevel clustering structure did not bias inferences and that the results are robust to alternative specifications.

Third, we conducted syndrome-level analyses across all eight TRF subscales to confirm that results were not confined to the broadband composites. Significant changes were detected for anxious/depressed, withdrawn, rule-breaking, and aggressive behavior syndromes, as reported above. Other syndromes (somatic complaints, social problems, thought problems, and attention problems) showed no significant temporal change. Applying false discovery rate (FDR) control (Benjamini–Hochberg, *q* < 0.05) confirmed that all significant syndrome-level changes remained significant, and no additional scales reached corrected thresholds. This suggests that the observed patterns are domain-specific rather than artifacts of multiple testing.

Finally, because scalar invariance was only partially achieved (two intercepts were freed), we repeated the GMM analyses using factor scores derived exclusively from invariant items. The three-class solutions for both internalizing and externalizing remained the best-fitting according to BIC (internalizing BIC = 2969.2; externalizing BIC = 3014.1) and entropy values remained acceptable (≥0.80). The size and characteristics of the low-stable, transient-elevated, and high-persistent classes were unchanged within rounding error. This provides additional confidence that minor departures from full invariance did not affect the trajectory classification results.

## 4. Discussion

The present study examined whether adolescents’ emotional and behavioral health change during a short-term study-abroad sojourn and whether distinct subgroups follow different adjustment trajectories. At the mean level, internalizing problems (anxious/depressed, withdrawn) increased from pre-departure to mid-sojourn and then remained elevated one month after return, whereas externalizing problems (rule-breaking, aggression) followed an inverted-U pattern, peaking mid-sojourn and returning near baseline post-return. Person-centered analyses further revealed three latent classes—low-stable, transient-elevated, and high-persistent—replicated across internalizing and externalizing domains. Taken together with measurement invariance and sensitivity checks, these results suggest that the sojourn functions as a moderate, time-bound stressor for most adolescents, while a small subgroup with pre-existing vulnerabilities shows sustained difficulties.

The mean trends are consistent with acculturation frameworks that anticipate a dip in adjustment following relocation (Neto, 2009). The rise in internalizing and externalizing symptoms at mid-sojourn dovetails with the “culture-shock” phase (H. J. Kim & Okazaki, 2014) and with transactional views of stress and adaptation (Golub et al., 2007). Similar short-term increases in distress among adolescent sojourners have been documented in prospective designs, and meta-analytic work also notes early elevations in strain (Aresi et al., 2019).

A notable asymmetry emerged post-sojourn: externalizing scores declined to baseline, but internalizing symptoms remained above pre-departure levels. This pattern is compatible with the “W-curve” or reentry difficulty posited for return transitions (González-Roz et al., 2024), and with reports that reentry can be as challenging as initial entry (Ormel et al., 2015). Behaviorally, rule-breaking and antagonism normalize quickly when adolescents re-enter familiar family and school structures, whereas anxious mood, rumination, and withdrawal often require more time. The lack of decline in internalizing symptoms between T2 and T3 may reflect lagged recovery and added stressors of reintegration (academic catch-up, renegotiating peer networks).

Our measurement schedule also helps explain timing. We assessed at pre-departure (T1), mid-sojourn (T2), and one month post-return (T3). Studies capturing the immediate arrival window sometimes report a brief honeymoon relief before later increases (Beukema, 2o2.) Because our first in-country measurement occurred mid-sojourn—after novelty had likely worn off—we may have captured the period of maximal disequilibrium. The magnitude of mean changes (~5 T-score points) suggests a moderate effect, salient but not indicative of widespread clinical deterioration, consistent with multi-country work showing modest average fluctuations amid individual variability (Corney et al., 2024).

Moving beyond averages, the three-class solutions align with person-centered evidence that intercultural transitions yield prototypical pathways (see also resilience typologies in the broader stress literature). Roughly two-thirds to four-fifths of adolescents followed a low-stable course, ≈17–28% showed a transient-elevated pattern, and a small high-persistent subset (≈5–7%) began elevated, worsened abroad, and remained high. These classes map closely onto the “resilient,” “recovery,” and “chronic distress” trajectories reported by Golub et al. (2007), with one difference: we did not detect an “improver” class. Age and method may explain this: our participants were mid-adolescents, and teacher ratings may not capture internal self-efficacy changes. Adolescents with high baseline symptoms may also lack resources to convert the sojourn into growth without support.

The class structure carries practical weight. The low-stable majority suggests that short-term exchanges are not inherently destabilizing. The transient-elevated group exemplifies the “stress-adaptation” sequence (Neto, 2010), where distress peaks and recovers; for these youths, front-loaded psychoeducation and mid-sojourn check-ins may hasten recovery. The high-persistent group highlights concentrated risk among adolescents with pre-existing problems; for them, separation from supports and adaptation demands likely exceeded coping capacity. Pre-departure screening and enhanced scaffolding (e.g., counseling, structured contact with home, host school coordination) are warranted.

Our externalizing inverted-U mirrors accounts of temporary increases in rule-breaking as adolescents learn new norms (Sung et al., 2019). That these behaviors returned to baseline by T3 suggests they are situational and responsive to structure. By contrast, persistent internalizing symptoms diverge from university studies, where well-being often normalizes after 3–6 months. Two factors likely explain this discrepancy. First, our T3 was only one month post-return; longer follow-up may have shown recovery. Second, developmental stage: secondary-school students return to parent- and school-regulated contexts with academic remediation demands and dense peer networks. Reentry tasks may maintain anxious or depressive affect even as behavior normalizes. This interpretation accords with reentry models emphasizing the “shock of the familiar” (Aresi et al., 2019).

Another divergence is the absence of an early “honeymoon” decrease. Measurement timing likely explains this difference, as our T2 fell mid-sojourn. Compared to the five-class stress patterns identified by previous studies, our three-class solutions are more parsimonious, reflecting fewer time points, teacher-report focus, and a homogeneous sample. Nonetheless, the core typologies—resilience, transient elevation, persistent elevation—are shared.

The differentiated post-sojourn trajectories reflect distinct determinants of emotional versus behavioral dysregulation. Externalizing problems are sensitive to contingencies and supervision; reentry to familiar rules quickly dampens acting-out. Internalizing problems are more tied to cognitive–affective processes—worry, negative biases, loneliness—that take longer to resolve and may be re-triggered by reentry demands. Moreover, study-abroad experiences can prompt identity work; returning adolescents sometimes report “in-between” feelings, which can sustain low mood or withdrawal. From a self-regulation perspective, behavioral regulation resynchronizes quickly with structure, while affective regulation needs deliberate support and time (Cerniglia et al., 2020; Cimino et al., 2020).

Threshold transitions and reliable change indices support these interpretations. About 10% more adolescents crossed into borderline/clinical internalizing range at T2 (two-thirds remitted by T3), while externalizing elevations largely remitted. Mapping these shifts onto latent classes confirmed that the transient-elevated subgroup accounted for most temporary clinical movement, whereas the high-persistent subgroup concentrated sustained cases. The sensitivity analysis excluding baseline-elevated adolescents nearly eliminated the high-persistent class, underscoring baseline risk as the key marker for sustained difficulty. This aligns with selective prevention principles: brief pre-departure screening can identify youths needing tailored supports. At the same time, our results caution against pathologizing normative, time-limited spikes: for many adolescents, mid-sojourn distress resolves naturally.

Programmatically, three levers emerge. Timing: concentrate monitoring and supports mid-sojourn when risk peaks, and extend into the first month post-return. Targeting: use pre-departure checklists to flag elevated students; coordinate scaffolding with host schools/families. Framing: normalize transient difficulties while providing concrete coping tools. For the small high-risk group, structured collaboration among schools, families, and clinicians can prevent escalation.

The study advances the literature with a prospective, three-wave design in secondary-school sojourners, a less-studied population. Use of a standardized instrument (TRF) with documented invariance supports inference about true change. Multilevel modeling addressed clustering, and results held under robustness checks. Growth mixture modeling identified meaningful heterogeneity, and linking class membership to thresholds enhanced applied relevance. Finally, findings replicate earlier descriptive work while extending it with trajectory classes and clinical movement estimates.

Limitations should be noted. Reliance on teacher report may underestimate internal states; multi-informant designs are preferable. Our post-return window was one month; longer follow-up is needed. The sample comprises Italian adolescents with uniform program length, limiting cultural generalizability. Mechanisms and predictors (stress, coping, proficiency) were not directly measured. The high-persistent class was small, so subgroup estimates must be interpreted cautiously. Finally, the three-wave design constrains trajectory shape; denser sampling would better resolve discrepancies regarding honeymoon and recovery phases. Moreover, findings should be interpreted considering the non-representative volunteer sample and the absence of formal longitudinal SEM/path modeling, which future work will address.

## 5. Conclusions

In summary, this study contributes to the literature by documenting both the average and divergent mental health trajectories of adolescents before, during, and after a study-abroad sojourn. The results largely reinforce prior findings and theoretical models of acculturative stress, while also highlighting the importance of individual differences. Most adolescents adapt well to the challenges of cultural immersion, but a notable subset experience meaningful (if temporary) distress, and a few carry significant risk that merits attention. By comparing these outcomes with previous studies, we found more similarities than differences—a testament to the robustness of cultural transition effects—and we offered hypotheses for the few discrepancies (such as timing of recovery). Understanding these patterns is not only of scholarly interest but also of practical significance: it can guide the development of screening tools, support services, and policies to ensure that international study experiences are as positive and growth-promoting as intended, with minimal adverse psychological consequences for young travelers. This study advances understanding of adolescent adjustment during short-term study-abroad by highlighting that pre-existing dysregulation may predict later maladjustment. Future research should examine whether parental scaffolding moderates these trajectories. Practical implications include developing screening and preventive interventions within school exchange initiatives

## Figures and Tables

**Table 1:** Descriptive statistics for psychopathological risk indicators at each time point (teacher-reported T-scores), with tests of mean differences over time.

Measure (T-Score)	T1 Pre-Departure M (SD)	T2 Mid-Sojourn M (SD)	T3 Post-Return M (SD)	T1–T2 Change (*p*)	T2–T3 Change (*p*)
Internalizing Problems	50.1 (9.4)	54.8 (10.5)	55.0 (10.2)	+4.7 (<0.001)	+0.2 (0.78)
Anxious/Depressed	54.0 (8.8)	58.9 (9.7)	58.0 (9.5)	+4.9 (<0.001)	−0.9 (0.40)
Withdrawn/Depressed	51.2 (8.1)	53.8 (8.4)	53.3 (8.6)	+2.6 (0.02)	−0.5 (0.50)
Externalizing Problems	48.5 (8.7)	53.2 (10.1)	49.7 (9.0)	+4.7 (<0.001)	−3.5 (<0.001)
Rule-Breaking Behavior	50.0 (9.5)	56.1 (10.8)	52.3 (9.9)	+6.1 (<0.001)	−3.8 (0.004)
Aggressive Behavior	47.3 (7.9)	50.2 (8.5)	46.1 (7.4)	+2.9 (0.01)	−4.1 (0.03)

**Table 2:** (**A**) Latent trajectory classes for (TRF T-scores). Class proportions, 95% confidence intervals, and mean trajectories are shown. (**B**) Latent trajectory classes for externalizing problems (TRF T-scores).

**(A)**					
**Latent Class**	**% of Sample (95% CI)**	**Mean T1**	**Mean T2**	**Mean T3**	**Trajectory Description**
Low-Stable	65% (59–71%)	45	48	46	Low stable emotional symptoms (normal range throughout)
Transient-Elevated	28% (22–34%)	51	60	53	Temporary spike into borderline range, recovery by T3
High-Persistent	7% (4–10%)	64	70	66	Elevated at baseline, worsening at T2, still high at T3
**(B)**					
**Latent Class**	**% of Sample (95% CI)**	**Mean T1**	**Mean T2**	**Mean T3**	**Trajectory Description**
Low-Stable	78% (72–83%)	44	47	45	Consistently low behavioral problems (normal range throughout).
Transient-Elevated	17% (12–22%)	50	61	51	Temporary spike into borderline during sojourn, returns to near the baseline post-return.
High-Persistent	5% (3–8%)	63	72	65	Elevated at baseline, further increase mid-sojourn, still high after return.

(**A**) Note. T-score means were model-estimated class means (rounded). Class percentages reflected average posterior probabilities. Borderline ≈ T ≥ 60; clinical ≈ T ≥ 64. A three-class growth mixture model was retained on fit and parsimony (BIC = 2968.0; entropy = 0.84; Lo–Mendell–Rubin-adjusted LRT, k = 3 vs. 2: *p* = 0.003; k = 4 vs. 3: *p* = 0.11). Longitudinal comparability was supported by (partial) scalar invariance across T1–T3. (**B**) Note. T-score means were model-estimated class means (rounded). Borderline ≈ T ≥ 60; clinical ≈ T ≥ 64. Three-class GMM was retained based on fit and parsimony (BIC = 3012.7; entropy = 0.81; Lo–Mendell–Rubin-adjusted LRT, k = 3 vs. 2: *p* = 0.015; k = 4 vs. 3: *p* = 0.19).

## Data Availability

Data will be available via request to the authors. During the preparation of this paper, the authors employed ChatGPT 4.0 to enhance linguistic clarity, readability, rectify grammatical errors, and refine academic expressions in non-native English sections of the manuscript. The AI-generated suggestions were meticulously reviewed, modified, and validated by the authors to ensure adherence to scholarly standards. The authors confirm that no AI-generated interpretations, conclusions, or data analyses were incorporated into the final content, and they assume full responsibility for the accuracy and originality of the research.
